# miR‐18a promotes *Mycobacterial* survival in macrophages via inhibiting autophagy by down‐regulation of ATM

**DOI:** 10.1111/jcmm.14899

**Published:** 2019-12-17

**Authors:** Qiulu Yuan, Haotian Chen, Yuxin Yang, Yurong Fu, Zhengjun Yi

**Affiliations:** ^1^ Key Laboratory of Clinical Laboratory Diagnostics in Universities of Shandong Department of Laboratory Medicine Weifang Medical University Weifang China; ^2^ Department of Medical Microbiology Clinical Medicine College Weifang Medical University Weifang China

**Keywords:** ataxia telangiectasia mutated, autophagy, exosome, miR‐18a, *Mycobacterium tuberculosis*

## Abstract

Tuberculosis (TB), caused by *Mycobacterium tuberculosis* (Mtb), is one of leading causes of global deaths. This study aimed to explore the role of miR‐18a in RAW264.7 cells response to Mtb infection. Exosomes derived from Mtb‐infected cells were isolated and further validated by size, transmission electron microscopy and Western blot. RT‐PCR was utilized to measure miR‐18a expression. Cell viability and ultrastructure were examined by CFU counting, CCK‐8 and electron microscope, respectively. Potential target genes of miR‐18a were predicted with bioinformatics and further confirmed using RT‐PCR, Western blot and laser confocal microscope analysis, respectively. LC3, AMPK and mTOR were measured using Western blot. We found that miR‐18a was induced both in Mtb‐infected RAW264.7 cells and its derived exosomes compared with the controls. In addition, up‐regulation of miR‐18a promoted intracellular Mtb survival, attenuated cell viability and reduced LC3‐II level, while its down‐regulation had the opposite effect. miR‐18a overexpression suppressed level of ATM, one possible target of miR‐18a, while its underexpression enhanced ATM. We also found that inhibition of ATM induced LC3‐II decrease in Mtb‐infected cells and could reverse the increase of LC3‐II caused by inhibition of miR‐18a. Moreover, down‐regulation of miR‐18a increased p‐AMPK level while reduction of ATM could reverse the change. Taken together, our results suggest that miR‐18a is up‐regulated in macrophages response to Mtb infection, and it promotes intracellular Mtb survival through repressing autophagic process by down‐regulation of ATM pathway. This provides new thought for TB pathogenesis, diagnosis and treatment.

## 1 INTRODUCTION

Tuberculosis (TB), a respiratory disease caused by *Mycobacterium tuberculosis* (Mtb), is one of the most deadly infectious diseases in the world.^1^ Macrophages are the main target cells of Mtb and are also the main first‐line defence against TB in vivo.^2^ It is estimated that 40%‐70% of infected people terminate infection caused by Mtb only by innate immune mechanisms.^3^ The establishment of infection depends on the ability of Mtb survival in macrophages and the multiple interactions between Mtb and host cells, thereby determining the outcome of the infection.^4^

microRNA (miRNA) is an endogenous, non‐coding small RNA, which plays a significant role in regulating gene expression at the post‐transcriptional level, including host immune responses.^5^, ^6^ It has been reported that many miRNAs, such as miR‐125, miR‐144 and miR‐155, are involved in modulation of innate immunity and adaptive immunity including B cell differentiation, antibody production as well as T cell development.^7^, ^8^, ^9^, ^10^ Moreover, growing evidence has suggest that many miRNAs play important regulatory roles in the immune response against TB.^11^, ^12^, ^13^, ^14^, ^15^

miR‐18a is a member of the miR‐17 family, which encodes for six individual miRNAs including miR‐17, miR‐18a, miR‐19a, miR‐20a, miR‐19b and miR‐92a.^16^ Studies have shown that many of them are related to Mtb infection: miR‐17‐5p regulates autophagy by targeting Mcl‐1and STAT3 in Mtb‐infected RAW264.7 cells, miR‐20a inhibits autophagy by targeting ATG16L1 and ATG7 to facilitate BCG survival in macrophages, and miR‐92a is increased in serum from pulmonary TB patients.^17^, ^18^, ^19^ Many studies have reported that miR‐18a, the most prominent miRNA in the miR‐17‐92 family, is up‐regulated in activated T cells, can activate rapamycin‐induced autophagy and is involved in regulation of autophagy in colon cancer cells.^20^, ^21^ However, little is known about the impact of miR‐18a on Mtb survival and its possible underlying mechanism. In the current study, we first investigated the role of miR‐18a in macrophages response to Mtb infection.

## 2 MATERIALS AND METHODS

### 2.1 Infection of RAW264.7 cells with Mtb

Mtb strain H37Rv, grown on Lowenstein‐Jensen (LJ) medium, was collected and then dispersed into single bacterial suspension in RPMI 1640 by vortex and needle aspiration, which was further confirmed by acid‐fast staining. RAW264.7 cells were cultured in RPMI 1640 medium added with 10% foetal bovine serum in an incubator with 5% CO_2_ at 37°C. Cells were infected with single Mtb at the indicated multiplicity of infection (MOI = 1, 5 and 10) and further cultured for required time period (6, 12, 24 and 48 hours).

### 2.2 Preparation of exosome

RAW264.7 cell culture supernatant was harvested at indicated time‐points post‐infection (6, 12, 24 and 48 hours), and exosome was isolated using PureExo^®^ Exosome Isolation kit (101Bio). Exosomal size was detected with Malvern Zetasizer Nano (Malvern Panalytical) and transmission electron microscopy (TEM), respectively. Moreover, CD63, one known exosomal marker, was measured by Western blot.

### 2.3 RT‐PCR

Total RNA was extracted from RAW264.7 cells or its derived exosomes using TRIzol reagent (Invitrogen), and cDNA was then synthesized by cDNA Synthesis Kit (Takara La Taq). Polymerase chain reaction (PCR) was run under the cycling conditions for miR‐18a: 95°C for 5 minutes, followed by 35 cycles of 95°C for 10 seconds, 60°C for 20 seconds and 72°C for 20 seconds, and for ataxia telangiectasia mutated (ATM): 95°C for 4 minutes, followed by 30 cycles of 95°C for 10 seconds, 58°C for 20 seconds, 72°C for 60 seconds and 72°C for 5 minutes. U6 or β‐actin was used as internal controls. The primers used above were shown in Tables S1 and S2.

### 2.4 Evaluation of the effect of miR‐18a on Mtb survival in RAW264.7 cells

RAW264.7 cells were transfected with miR‐18a mimics, mimics NC, miR‐18a inhibitor or inhibitor NC (GenePharma) with Lipofectamine 2000 (Invitrogen). FAM‐siRNA (GenePharma) was used to detect transfection efficiency. Following transfection, cells were then infected with Mtb (MOI = 10). Cells were lysed with 0.5% TritonX‐100 for 20 minutes at 24 hours after infection, and resulted lysate was cultured on LJ medium for colony‐forming unit (CFU) counting. Meantime, cells were seeded on cover slides in 24‐well plates for acid‐fast staining.

### 2.5 Cell viability assay and ultrastructural analysis

After transfection and following infection with Mtb at MOI = 10 for 6, 12, 24 and 48 hours, the cell counting kit‐8 reagent of 10 μL (CCK‐8; Dojindo) was added into each well and continued to culture for 1.5 hours. The OD value was detected by spectrophotometer (Thermo Fisher Scientific Oy) at 450 nm wavelength. Simultaneously, RAW264.7 cells were collected and fixed in glutaraldehyde for ultrastructure analysis under an electron microscope.

### 2.6 Western blot analysis

Protein samples were electrophoresed on sodium dodecyl sulphate‐polyacrylamide gel electrophoresis (SDS‐PAGE) and transferred to polyvinylidene difluoride (PVDF) membrane. The membranes were then incubated with anti‐LC3 (Abcam), anti‐ATM (Abcam), anti‐pAMPK/AMPK (Abcam), anti‐pmTOR/mTOR (Abcam) or anti‐CD63 (Sangon Biotech). eECL kit (Beyotime) was used to illuminate, and the Chemi‐Doc XRS+ (Bio‐Rad) was used to imaging of membranes. β‐tubulin was used as internal control.

### 2.7 Prediction and confirmation of potential target gene ATM of miR‐18a

Possible target genes of miR‐18a were predicted by the TargetScan software. Then, the complementary sites and pairing scores between miR‐18a and putative target ATM were analysed by TargetScan. In addition, the correlation analysis was performed between miR‐18a expression and ATM mRNA expression in Mtb‐infected RAW264.7 cells. The effect of modulation of miR‐18a on ATM expression was examined by RT‐PCR and Western blot. Afterwards, levels of pAMPK and pmTOR were evaluated for illustrating the potential role of miR‐18a in the ATM‐AMPK‐mTOR autophagy pathway.

### 2.8 Confocal microscopy assay

RAW264.7 cells were fixed with 4% paraformaldehyde for 20 minutes and then treated with 0.5% TritonX‐100 for 10 minutes. After being blocked with 5% bovine serum albumin (BSA) for 30 minutes, cells were incubated with anti‐ATM followed by Cy3‐labelled goat anti‐rabbit IgG (Boster Biological Technology) and were further stained with DAPI for 5 minutes. Cells were observed under confocal laser scanning microscope (OLYMPUS).

### 2.9 Statistical analysis

All the data were displayed as mean ± standard deviation (SD) of three independent experiments. The Student's *t* test was used to compare two groups, and the one‐way ANOVA was used to compare multiple samples. Significant statistical differences were deemed to *P* < .05.

## 3 RESULTS

### 3.1 Mtb infection induced miR‐18a overexpression both in RAW264.7 cells and its derived exosomes

**Figure 1:**
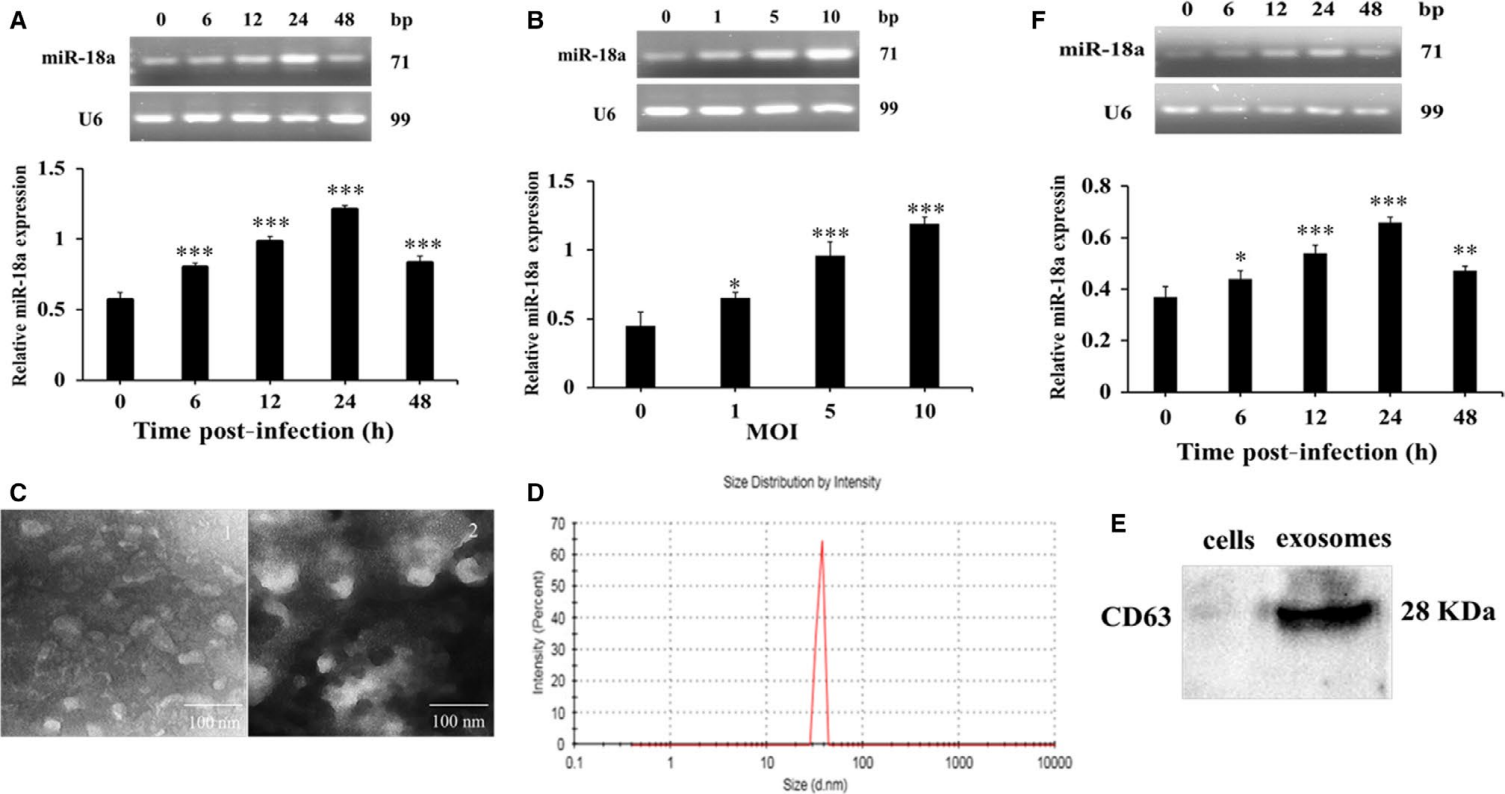
The expression of miR‐18a was induced after Mtb infection. A, The expression of miR‐18a at different time‐points in RAW264.7 macrophages infected by Mtb. B, The expression of miR‐18a at different MOI in RAW 264.7 macrophages infected by Mtb. C, Observation of exosomal characteristic by TEM. D, Estimation of the exosomal diameter by particle size analysis. E, Detection of the exosomal protein marker CD63 by Western blot. F, The expression of miR‐18a at different time‐points in exosomes derived from Mtb‐infected RAW264.7 macrophages. Data represent the means ± SD from three independent experiments. *P < .05, **P < .01, ***P < .001. Relative miR‐18a expression, miR‐18a/ internal reference U6

In order to evaluate the effect of Mtb infection on miR‐18a expression in macrophages, we assessed its level in Mtb‐infected RAW264.7 cells. The results showed that intracellular miR‐18a expression displayed a MOI‐ and time‐dependent elevation upon Mtb infection (Figure 1A,B). RAW264.7 cell‐derived exosomes were successfully obtained based on the confirmation by TEM, particle size and marker protein CD63 analysis (Figure 1C‐E). We also found that miR‐18a was gradually up‐regulated in the exosomes derived from Mtb‐infected RAW264.7 cells in a time‐dependent manner (Figure 1F). The data demonstrated that levels of both intracellular and exosomal miR‐18a were mightily induced, which suggested that miR‐18a may play pivotal role in the pathogenesis of TB.

### 3.2 miR‐18a facilitated Mtb survival in RAW264.7 cells

**Figure 2:**
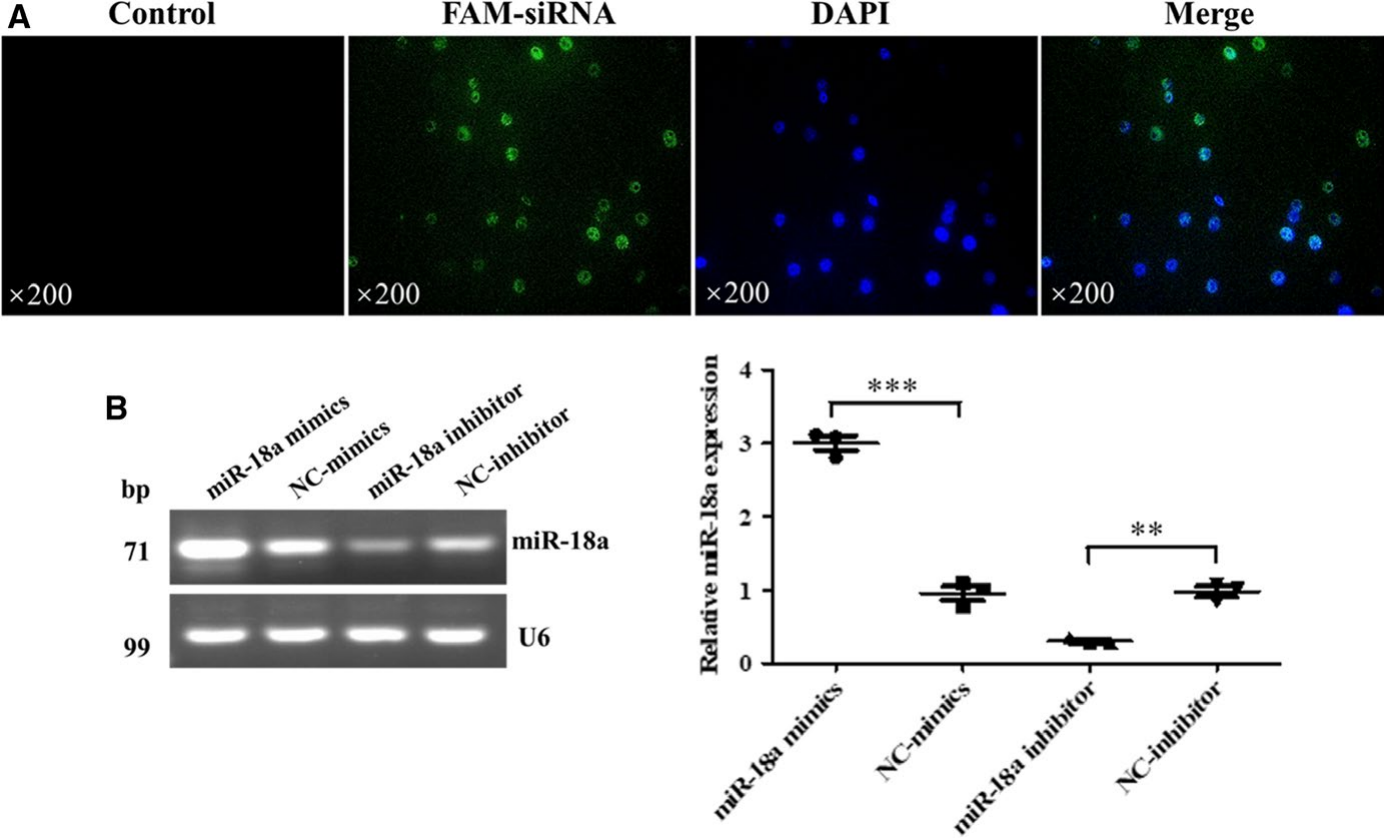
Identification of models of overexpression and inhibit expression of miR‐18a. A, The transfection efficiency of RAW264.7 macrophages transfected with FAM‐siRNA was detected by fluorescence microscope. B, The expression of miR‐18a in RAW264.7 macrophages transfected with miR‐18a mimics, NC‐mimics, miR‐18a inhibitor or NC‐inhibitor by RT‐PCR. Data represent the means ± SD from three independent experiments. **P < .01, ***P < .001. Relative miR‐18a expression, miR‐18a/ internal reference U6

**Figure 3:**
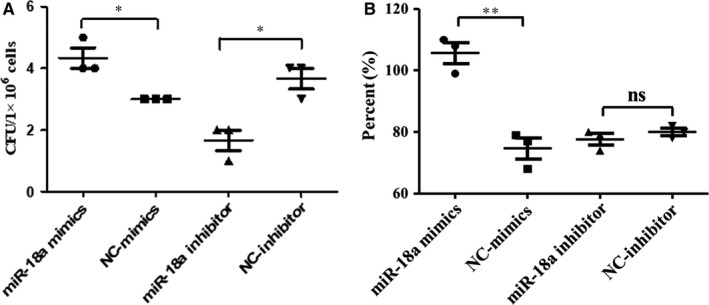
Effect of miR‐18a on the survival of Mtb in RAW264.7 cells. A, The effect of Mtb survival in RAW264.7 macrophages transfected with miR‐18a mimics, NC‐mimics, miR‐18a inhibitor or NC‐inhibitor was measured by CFU analysis. B, The effect of Mtb survival in RAW264.7 macrophages transfected with miR‐18a mimics, NC‐mimics, miR‐18a inhibitor or NC‐inhibitor was measured by acid‐fast stain. Data represent the means ± SD from three independent experiments. *P < .05, **P < .01. CFU, colony‐forming unit

Then, we further explored the impact of regulation of miR‐18a on intracellular Mtb survival. Modulation of miR‐18a in RAW264.7 cells was achieved with its mimics or inhibitor. RT‐PCR results showed that miR‐18a mimics significantly increased miR‐18a level and miR‐18a inhibitor decreased its level in RAW264.7 cells (Figure 2A‐B). Subsequently, we further determined whether miR‐18a exert an effect on intracellular Mtb survival using CFU and acid‐fast staining. As exhibition in Figure 3A, miR‐18a up‐regulation resulted in a significant increase while its down‐regulation led to an obvious decrease of CFU number, which were identical with the results of acid‐fast staining (Figure 3B). The above observations implicated that miR‐18a could promote Mtb survival within macrophages.

### 3.3 Effect of miR‐18a on Mtb‐infected macrophages

**Figure 4:**
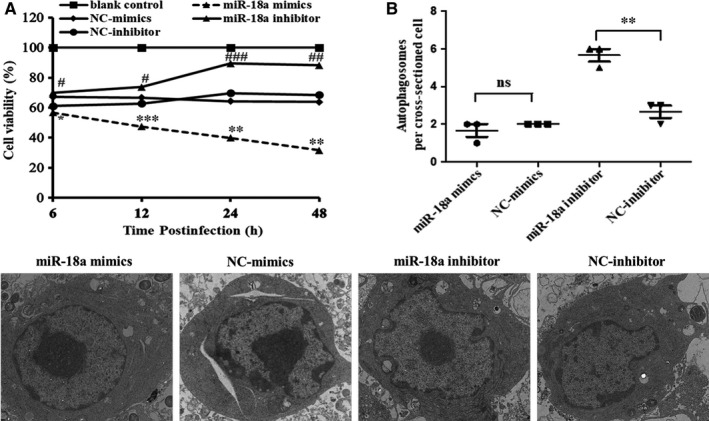
Effect of miR‐18a on the Mtb‐infected RAW264.7 macrophages. A, The viability of RAW264.7 cells was detected by CCK‐8. B, The ultrastructure changes of RAW264.7 macrophages were detected by TEM. The number of autophagosomes per cross‐sectioned cell was counted (20 cells per group counted by TEM). Data represent the means ± SD from three independent experiments. *P < .05, **P < .01, ***P < .001, ^#^ *P* < .05, ^##^ *P* < .01, ^###^ *P* < .001

We further studied the effect of miR‐18a on Mtb‐infected macrophages. Compared with the controls, overexpression of miR‐18a inhibited cells viability whereas underexpression of miR‐18a enhanced cells viability at 6, 12, 24 and 48 hours post‐Mtb infection, respectively (Figure 4A). The ultrastructure changes of RAW264.7 cells were observed by TEM, and as shown in Figure 4B, a number of autophagosomes were present in cells transfected with miR‐18a inhibitor, while few autophagosomes were observed in miR‐18a mimics treated ones. Therefore, we inferred that miR‐18a may be involved in the regulation of autophagy (Figure 4B).

### 3.4 miR‐18a suppressed autophagy in Mtb‐infected macrophages by inhibiting ATM

**Figure 5:**
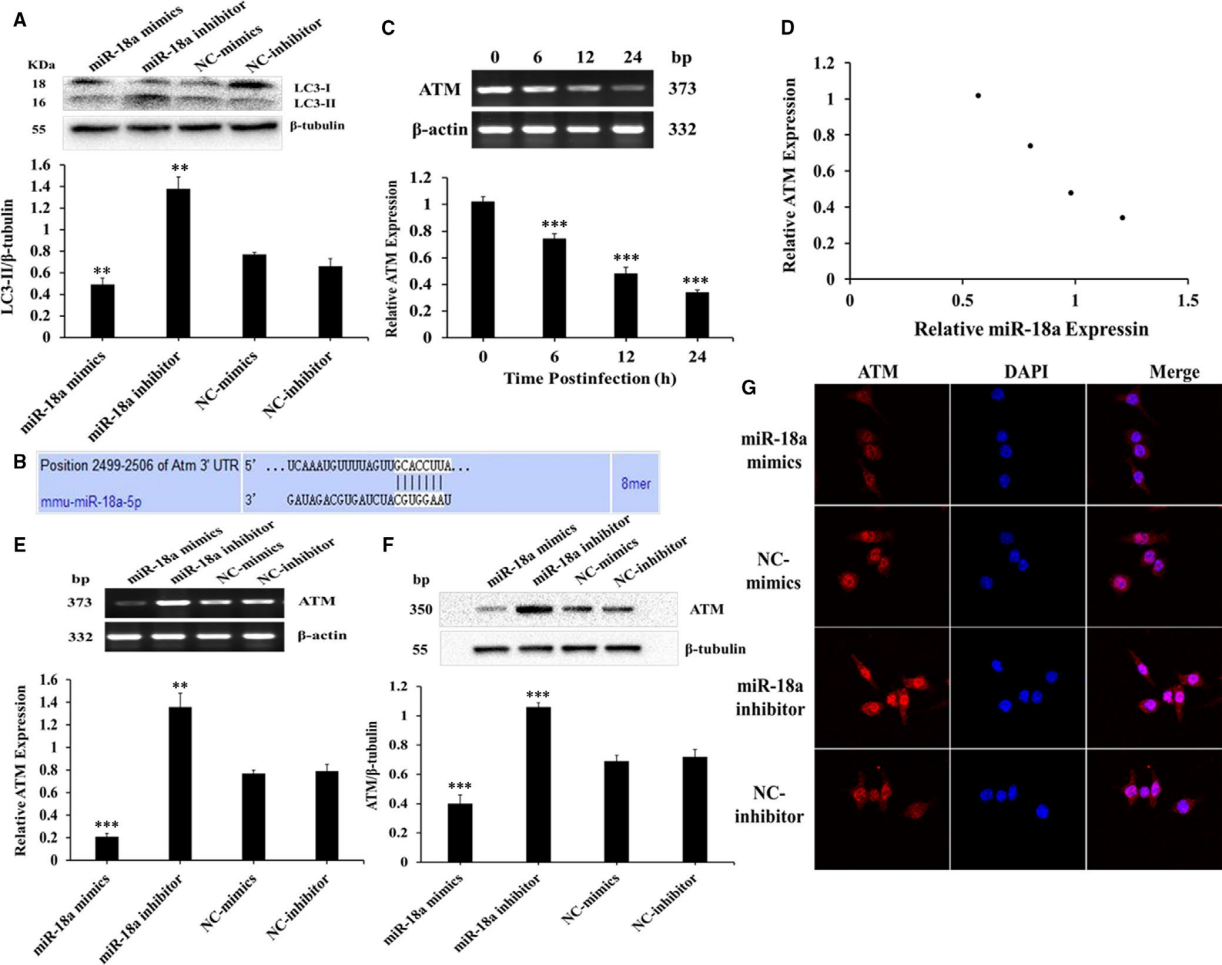
miR‐18a inhibits autophagy in RAW264.7 macrophages by suppressing ATM. A, The expression of LC3‐II in RAW264.7 macrophages transfected with miR‐18a mimics, NC‐mimics, miR‐18a inhibitor or NC‐inhibitor followed by Mtb infection. B, The binding sites and matching degree were predicted between miR‐18a and ATM by TargetScan analysis. C, The expression of ATM mRNA at different time‐points in RAW264.7 macrophages infected with Mtb by RT‐PCR. D, Correlation analysis between miR‐18a and ATM expression. E, The expression of ATM mRNA in RAW264.7 macrophages transfected with miR‐18a mimics, NC‐mimics, miR‐18a inhibitor or NC‐inhibitor followed by Mtb infection by RT‐PCR. F, The expression of ATM protein in RAW264.7 macrophages transfected with miR‐18a mimics, NC‐mimics, miR‐18a inhibitor or NC‐inhibitor followed by Mtb infection by Western blot. G, Observation of ATM immunofluorescence by laser scanning confocal microscope. Data represent the means ± SD from three independent experiments. **P < .01, ***P < .001. Relative ATM expression, ATM/β‐actin

In order to validate the impact of miR‐18a on autophagy, autophagy‐related protein LC3 was assessed by Western blot. Results showed that miR‐18a mimics significantly decreased while its inhibitor increased LC3‐II expression in Mtb‐infected RAW264.7 cells compared with the controls (Figure 5A). The data indicated that miR‐18a restrained autophagy in macrophages response to Mtb. Next, we furthered investigated the underlying autophagy mechanisms triggered by miR‐18a.

**Figure 6:**
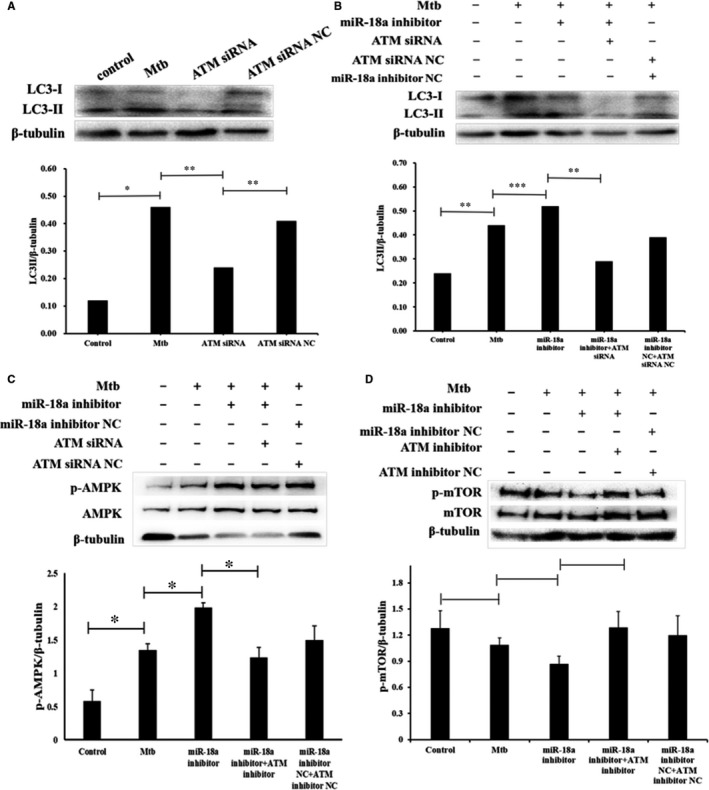
miR‐18a inhibited autophagy in RAW264.7 macrophages by ATM‐AMPK pathway. A, The RAW264.7 cells were infected with Mtb or without Mtb and then transfected with ATM siRNA or ATM siRNA NC. The expression of LC3‐II in RAW264.7 macrophages was determined by Western blot. B, The RAW264.7 cells were infected with Mtb or without Mtb and then transfected with miR‐18a inhibitor or ATM siRNA. The expression of LC3‐II in RAW264.7 macrophages was determined by Western blot. C, The RAW264.7 cells were infected with Mtb or without Mtb and then transfected with miR‐18a inhibitor or ATM siRNA. The expression of p‐AMPK in RAW264.7 macrophages was determined by Western blot. D, The RAW264.7 cells were infected with Mtb or without Mtb and then transfected with miR‐18a inhibitor or ATM siRNA. The expression of p‐mTOR in RAW264.7 macrophages was determined by Western blot. Data represent the means ± SD from three independent experiments. **P < .01, *P < .05

Target genes of miR‐18a were predicted by TargetScan (Table S3). ATM, a protein kinase, is known for its critical roles in activation of autophagy.^22^ We found that it was one potential target of miR‐18a (Figure 5B). However, the function of ATM in TB remains unknown until now. Hence, we decided to detect ATM expression in RAW264.7 cells response to Mtb infection. RT‐PCR data showed that ATM expression exhibited a significant downward trend in Mtb‐infected RAW264.7 cells at different time‐points, which was negatively correlated with the expression of miR‐18a (Figure 5C,D). In order to further verify the relationship between miR‐18a and ATM, RT‐PCR and Western blot were applied to detect the effect of modulation of miR‐18a on ATM. As present in Figure 5E,F, miR‐18a mimics significantly down‐regulated while miR‐18a inhibitor up‐regulated ATM expression in Mtb‐infected cells compared with the controls, respectively. Consistently, the same was true of immunofluorescence observed by laser confocal microscope (Figure 5G). The above results indicated that miR‐18a could negatively modulate ATM expression, and together based on the roles of ATM in autophagy documented by many studies, we therefore inferred that ATM may be involved in autophagy process regulated by miR‐18a in Mtb‐infected macrophages. We then evaluated the effect of ATM on autophagy in RAW264.7 cells response to Mtb infection. Down‐regulation of ATM significantly reduced autophagy in Mtb‐infected macrophages (Figure 6A) and could reverse activation of autophagy caused by inhibition of miR‐18a (Figure 6B). This suggested that miR‐18a inhibited autophagy by decrease of ATM in Mtb‐infected RAW264.7 cells. Studies have reported that AMPK and mTOR play important roles in regulating autophagy. Consequently, we further assessed the impacts of modulation of miR‐18a‐ATM on p‐AMPK and p‐mTOR. Inhibition of miR‐18a up‐regulated expression of p‐AMPK, while down‐regulating ATM could reverse the change (Figure 6C). However, there was no significant change for expression of p‐mTOR (Figure 6D). These indicates that miR‐18a can attenuate autophagy in Mtb‐infected macrophages via regulation of ATM‐AMPK pathway.

## 4 DISCUSSION

miRNAs are important regulators involved in immune responses and disease progression.^23^ Recent studies have demonstrated the critical role of miRNAs in the evasion of Mtb from autophagic clearance of macrophages.^24^ miR‐18a is a member of the miR‐17‐92 family which has been demonstrated to play an important role in regulating autophagy. However, the detailed mechanism of miR‐18a in TB remains unclear. In this study, we found that Mtb induced miR‐18a expression in macrophages, which indicates that miR‐18a may contribute to the pathogenesis of TB.

Exosomes are biologically active vesicles produced by most cells, 30‐100 nm in diameter, and their release can be stimulated by inflammation and hypoxia, which can reflect the abnormality of cells and the states of diseases.^25^ Moreover, it has been reported that exosomes isolated from Mtb‐infected macrophages and Mtb‐infected mice serum can promote both innate and acquired immune responses in vitro and in vivo, respectively.^26^ Furthermore, exosomes have been proved to be an effective tools for miRNA delivery.^27^ In the study, we also investigated miR‐18a expression in exosomes derived from macrophages response to Mtb infection. Our data revealed that consistent with the variation tendency in Mtb‐infected macrophages, miR‐18a in exosomes was also increased.

Macrophages are the first barrier to Mtb infection and its activation such as autophagy formation can decrease intracellular Mtb burden.^28^ In this study, we explored the effect of miR‐18a on Mtb survival in macrophages. Our results showed that there was a positive correlation between miR‐18a expression and Mtb load in macrophages. Next, we further explored the underlying mechanism of miR‐18a promotion of intracellular Mtb survival.

Autophagy is one of the effective ways for host cells to defend against Mtb, which can directly eliminate intracellular Mtb, thereby limiting the growth of Mtb.^29^ In the study, electron microscopic results exhibited that more autophagosomes were shown in miR‐18a inhibitor transfected cells compared with the controls, indicating that miR‐18a may be associated with autophagy. To further examine the regulatory role of miR‐18a in autophagy, we examined the impact of miR‐18a on expression of autophagic protein LC3. Underexpression of miR‐18a elevated LC3‐II level while its overexpression inhibited LC3‐II expression, which suggests that miR‐18a negatively regulates autophagy. It also led us to further investigate the role of miR‐18a in autophagy signalling pathway.

Studies have shown that ATM maintained the autophagy pathway by activating the LKB1/AMPK/TSC2 signalling pathway and suppressing the negative regulator mTORC1.^30^ Bioinformatic analysis shown that ATM might be an autophagy‐related gene that miR‐18a may target. Moreover, we found that there was a negative correlation between ATM expression and miR‐18a level in Mtb‐infected macrophages. Then, we investigated the relationship between them. Indeed, our data showed that up‐regulation of miR‐18a suppressed ATM expression, whereas its down‐regulation elevated ATM level. Next, we found that down‐regulation of ATM suppressed autophagy process in macrophage response to Mtb infection and could reverse autophagy activation elicited by inhibition of miR‐18a. These results imply that miR‐18a can negatively regulate autophagy by inhibition of ATM.

AMPK is well known as one key signalling molecule which controls autophagy signalling pathway. Hence, we further explored the role of miR‐18a in the ATM‐AMPK‐mTOR autophagy pathway. Inhibition of miR‐18a up‐regulated p‐AMPK expression while the change could be reversed by down‐regulation of ATM. Combined these results, miR‐18a could attenuate autophagy in Mtb‐infected macrophages by modulation of ATM‐AMPK autophagy pathway.

In short, our study showed that miR‐18a expression, including exosomal miR‐18a, is induced upon Mtb infection, and miR‐18a accelerates Mtb survival in macrophages via negatively regulating autophagic process by inhibiting ATM. To the best of our knowledge, it is the first study to explore the role and mechanism of miR‐18a in Mtb infection, and this provide new ideas and theoretical basis for diagnosis of TB and the treatment of TB patients.

## CONFLICT OF INTEREST

The authors have no conflict of interest to declare related to this article.

## AUTHOR CONTRIBUTIONS

YF and ZY designed the study. QY, HC and YY performed the experiments. YF, ZY, QY, HC and YY analysed the data. QY drafted the manuscript. YF and ZY revised the manuscript and supervised the work.

## Data Availability

Data sets and materials used and/or analysed during the current study are available in the manuscript itself.
