# Supplementary material for: miR‐18a promotes Mycobacterial survival in macrophages via inhibiting autophagy by down‐regulation of ATM
Source: J Cell Mol Med. 2019 Dec 17;24(2):2004–12. doi: 10.1111/jcmm.14899 (PMC6991191; doi:10.1111/jcmm.14899)
Supplement: Supplementary file 1 [file JCMM-24-2004-s001.doc]

**Supplementary Table 1**

| Name | Primer sequence (5′- 3′) |
| --- | --- |
| miR-18a | Stem‐loop primer: GTCGTATCCAGTGCAGGGTCCGAGGTATTCGCA CTGGA TACGACCTATCT |
|  | Forward: CGCGTAAGGTGCATCTAGTGC |
|  | Reverse: AGTGCAGGGTCCGAGGTATT |
| U6 | Stem‐loop primer: GTCGTATCCAGTGCAGGGTCCGAGGTATTCGCA CTGGATACGACAAAAAT |
|  | Forward: AGAGAAGATTAGCATGGCCCCTG |
|  | Reverse: AGTGCAGGGTCCGAGGTATT |

**Supplementary Table** 2

| Name | Primer sequence (5′- 3′) |
| --- | --- |
| ATM | Forward: ACAGATGTGTCGTTGCGTAGC |
|  | Reverse: TCAGCGGAAGTGGATTGTAAGC |
| β‐actin | Forward: TGGCACCACACCTTCTACAATG |
|  | Reverse: CGCTCGGTCAGGATCTTCATG |

**Supplementary Table 3** The target genes of miR-18a

| Genes | Site type | Context score | *PCT |
| --- | --- | --- | --- |
| Etv6 | 8mer | -1.15 | 0.8 |
| Hif1a | 7mer-m8 | -0.89 | 0.75 |
| Zfp367 | 8mer | -0.70 | 0.73 |
| **Atm** | 8mer | -0.57 | 0.44 |
| Xylt2 | 8mer | -0.49 | 0.41 |
| Orai3 | 7mer-m8 | -0.37 | 0.29 |
| Tex2 | 7mer-m8 | -0.28 | 0.24 |
| Notch2 | 8mer | -0.20 | 0.14 |

*PCT: the probability of conserved targeting.
